# Network pharmacology-based exploration identified the antiviral efficacy of Quercetin isolated from mulberry leaves against enterovirus 71 via the NF-κB signaling pathway

**DOI:** 10.3389/fphar.2023.1260288

**Published:** 2023-09-19

**Authors:** Tianrun Liu, Yingyu Li, Lumeng Wang, Xiaomeng Zhang, Yuxuan Zhang, Xuejie Gai, Li Chen, Lei Liu, Limin Yang, Baixin Wang

**Affiliations:** ^1^ School of Medicine, Jiamusi University, Jiamusi, China; ^2^ The Affiliated First Hospital, Jiamusi University, Jiamusi, China; ^3^ School of Medicine, Dalian University, Dalian, China

**Keywords:** mulberry leaves, Quercetin, network pharmacology, enterovirus type 71, NF-kB

## Abstract

**Introduction:** Mulberry leaf (ML) is known for its antibacterial and anti-inflammatory properties, historically documented in “Shen Nong’s Materia Medica”. This study aimed to investigate the effects of ML on enterovirus 71 (EV71) using network pharmacology, molecular docking, and *in vitro* experiments.

**Methods:** We successfully pinpointed shared targets between mulberry leaves (ML) and the EV71 virus by leveraging online databases. Our investigation delved into the interaction among these identified targets, leading to the identification of pivotal components within ML that possess potent anti-EV71 properties. The ability of these components to bind to the targets was verified by molecular docking. Moreover, bioinformatics predictions were used to identify the signaling pathways involved. Finally, the mechanism behind its anti-EV71 action was confirmed through *in vitro* experiments.

**Results:** Our investigation uncovered 25 active components in ML that targeted 231 specific genes. Of these genes, 29 correlated with the targets of EV71. Quercetin, a major ingredient in ML, was associated with 25 of these genes. According to the molecular docking results, Quercetin has a high binding affinity to the targets of ML and EV71. According to the KEGG pathway analysis, the antiviral effect of Quercetin against EV71 was found to be closely related to the NF-κB signaling pathway. The results of immunofluorescence and Western blotting showed that Quercetin significantly reduced the expression levels of VP1, TNF-α, and IL-1β in EV71-infected human rhabdomyosarcoma cells. The phosphorylation level of NF-κB p65 was reduced, and the activation of NF-κB signaling pathway was suppressed by Quercetin. Furthermore, our results showed that Quercetin downregulated the expression of JNK, ERK, and p38 and their phosphorylation levels due to EV71 infection.

**Conclusion:** With these findings in mind, we can conclude that inhibiting the NF-κB signaling pathway is a critical mechanism through which Quercetin exerts its anti-EV71 effectiveness.

## 1 Introduction

Hand, foot, and mouth disease (HFMD) stands as a prevalent childhood infectious ailment primarily attributed to more than 20 enteroviruses. The prognosis for HFMD is generally optimistic, characterized by self-limiting symptoms that typically abate within a week (Zhang et al., 2022). However, certain neurological complications such as encephalomyelitis, brainstem encephalitis, and aseptic meningitis (Gonzalez et al.,2019) can rapidly lead to neurogenic pulmonary edema (Wang et al.,2019) and, in severe cases, even death. Enterovirus type 71 (EV71)is the most common viral culprit behind these severe complications (Yang et al., 2022). Currently, no clinically effective drugs exist, and symptomatic treatment remains the primary approach (Liu et al., 2015). HFMD has experienced multiple outbreaks worldwide, posing significant public health concerns and imposing substantial life safety risks and economic burdens on many countries (Solomon et al., 2010).

Chinese medicine considers virus infections as the invasion of cold and malevolent energy into the body. When the body’s defense is weak, this energy can easily invade. However, if the body’s defense is strong, it can resist such invasion (Li et al., 2020). Traditional Chinese medicine, with a history spanning five thousand years, has been routinely used to treat pandemics and endemic diseases, forming a comprehensive theoretical system for the prevention and treatment of deadly epidemics, referred to as “plagues” in ancient China (Qi and Tang, 2021). Traditional Chinese medicine is widely employed for antiviral purposes (Wu t al. 2021) by restoring the body’s overall balance to counteract the harmful effects of viral infections (Chen and Ye, 2022). Numerous traditional Chinese medicines have been proven to possess antiviral properties (Guan et al., 2020; Kang et al., 2021; Lee et al., 2021; Cui et al., 2022) capable of directly acting on viruses and stimulating the immune system to induce interferon production, thereby indirectly inactivating viruses (Huang et al., 2022).

Mulberry leaves (ML), derived from the Moraceae plant, are known for their antibacterial and antiviral effects (Chen et al., 2021). However, their potential anti-EV71 virus effect remains unexplored. This study aimed to investigate the potential of mulberry leaves in EV71 virus infection by predicting key target genes and signaling pathways involved in ML-mediated antiviral mechanisms through network pharmacology, bioinformatics, *in vitro* experiments, and medical statistics.

## 2 Materials and methods

### 2.1 Screening active components and target genes of ML

To screen the active components and target genes of ML, we employed the traditional Chinese medicine database TCMSP (https://old.tcmsp-e.com/). The screening criteria were defined as oral availability (OB) ≥ 30 and drug-likeness (DL) ≥ 0.18 (Cui et al., 2021). These criteria allowed us to identify the critical active ML ingredients and retrieve their target information. The target information was standardized using the Uniprot database to obtain the Gene name. Finally, we employed Cytoscape (V3.9.1) software to visualize the network diagram illustrating the connections between the “Chinese medicine-active ingredient-target gene.”

### 2.2 Construction of a protein interaction network diagram for target genes in ML

For the analysis of target genes in ML, we employed the STRING database (https://string-db.org/). The biological species “*Homo sapiens*” was specifically chosen, and the minimum interaction score was set at a medium confidence level (0.400). The resulting protein-protein interaction (PPI) network diagram was thoroughly examined, and the Cytoscape (V3.9.1) software was utilized to enhance the clarity of the PPI network by emphasizing the interaction scores.

### 2.3 Retrieval of EV71 virus genes and screening of Anti-EV71 related genes in ML

The GENECARDS database (https://www.genecards.org) and NCBI database (https://www.ncbi.nlm.nih.gov) were queried using “EV71”to retrieve the relevant genes associated with the EV71 virus. With the help of the VENN graph, we compared target genes of ML and EV71 viruses to identify potential anti-EV71 genes for ML. (http://jvenn.toulouse.inra.fr/app/example.html). Cytoscape (V3.9.1) was used to visualize potential target genes and their corresponding active components.

### 2.4 Molecular docking

Based on the research mentioned above, we identified the main components of ML and its target genes. The top ten target genes were retrieved from the PDB protein database (https://www.rcsb.org/) in PDB format, using “*Homo sapiens*” and “A≤2.5”as the specified settings. These PDB files underwent preprocessing, including water and residue removal, using the MOE 2019.0102 software. The CID numbers corresponding to the top ten active ingredients with antiviral effects in ML were obtained from the TCMSP database. These CID numbers were then retrieved from the PubChem database to retrieve the SDF file containing their 3D molecular structures (https://pubchem.ncbi.nlm.nih.gov/). Subsequently, utilizing the MOE 2019.0102 software, a small molecule library was constructed based on the obtained SDF files. Finally, molecular docking was performed between the processed macromolecule receptors and the ligands in the small molecule library.

### 2.5 GO functional enrichment and KEGG pathway analysis

An analysis was conducted using the DAVID database to explore the potential genes implicated in the anti-EV71 activity of Quercetin, the primary component of ML (https://david.ncifcrf.gov/home.jsp). Enrichment analysis of Gene Ontology (GO) functions and KEGG signaling pathways was performed, considering a significance threshold of *p* < 0.05. The top 15 enriched GO functions and KEGG signaling pathways were selected based on the obtained *p* values. The resulting enriched information was visualized on the MICROBIOTIC website (http://www.bioinformatics.com.cn/).

### 2.6 *In vitro* experimental study of quercetin against EV71 virus

#### 2.6.1 Cytotoxicity assay of quercetin and virus TCID_50_ assay

A stock solution of Quercetin (Sigma-Aldrich Cat. No.: CAS6151-25-3) was meticulously prepared at a concentration of 200 mM in DMSO, subsequently undergoing filtration through an organic microporous membrane. Human rhabdomyosarcoma (RD) cells were uniformly seeded within 96-well plates, achieving a density of 2 × 105 cells/mL. Each well received 100 μL of the cell suspension. To minimize the cytotoxicity of DMSO, it was diluted at a ratio of 1:1000. Quercetin stock solution was further diluted using 10% FBS in DMEM (Nissui Cat. No.: 05900) to a maximum concentration of 200 μM, with subsequent equal-fold dilutions. A 200 μL volume of the diluted solution was introduced into each well, where a monolayer of RD cells had been established. The cells were subsequently incubated at 37°C with 5% CO_2_ for 24, 48, and 72 h. Cell viability assessment was performed using the CCK8 assay (Beyotime Cat. No.: C0039), aimed at identifying the non-toxic concentration (TC0) of Quercetin concerning RD cells. The EV71 virus was diluted with a 2% FBS maintenance solution (Beyotime Cat. No.: C0232) and used to infect RD cells at concentrations ranging from 10^−1^ to 10^−8^. The TCID_50_ of the EV71 virus was calculated using the Reed-Muench method.

#### 2.6.2 Evaluation of Quercetin’s inhibitory effect on EV71 virus-infected RD cells

To assess the impact of Quercetin on EV71 virus-infected RD cells, we determined the maximum non-toxic concentration of Quercetin and prepared dilutions in equal increments. RD cells were infected with 100 TCID50 of the virus as the infection concentration. Quercetin concentrations of 25, 12.5, 6.25, and 3.125 μM were prepared using DMEM with 2% FBS as the solvent. Cells were treated with 100 μL of the EV71 virus and Quercetin simultaneously for 2 h. Subsequently, the Quercetin and virus were removed, the cells were washed once with PBS, and 200 μL of DMEM containing 2% FBS was added. The inhibitory effect of Quercetin on the EV71 virus was assessed at 48 h using the CCK-8 assay. In addition, varying concentrations of Quercetin were co-administered with EV71 to RD cells. After 48 h, the viral supernatant was harvested and underwent three freeze-thaw cycles to release EV71 from the RD cells. Subsequently, the mixture was centrifuged at 3,500 rpm for 15 min, and the supernatant was collected. Finally, the TCID50 value of the EV71 virus was quantified in the presence of distinct concentrations of Quercetin.

#### 2.6.3 Immunofluorescence analysis of Quercetin’s effect on EV71 virus-infected RD cells

RD cells were seeded at 1 × 105 cells/mL concentration on 24-well plates and specialized cell slides. Upon reaching 80% confluency, a concentration of Quercetin, known for its significant anti-EV71 effect, was added to the cells, along with 100 TCID50 of EV71. After 48 h, the cells were fixed with 4% paraformaldehyde for 15 min and permeabilized with 0.2% TritonX-100 (diluted in PBS) for 10 min at room temperature. A blocking solution containing 1% BSA, 3% donkey serum, and 0.1% TritonX-100 in PBS was applied at room temperature for 45 min. The primary antibodies (VP-1, Invitrogen Cat. No.: WD3250882A, Abnova Cat. No.: MAB1255-M08, p-NF-κB p65, Santa Cruz Cat. No.: sc-135769, TNF-α, Cell Signaling Cat. No.: D2D4, IL-1β, Santa Cruz Cat. No.: sc-52012) diluted in blocking buffer were added and incubated overnight at 4°C. The secondary antibodies (Alexa Fluor 488-labeled Goat Anti-Rabbit IgG and Alexa Fluor 488-labeled Goat Anti-Mouse IgG, Beyotime Cat. No.: A0423, A0428, (Alexa Fluor 594-labeled Goat Anti-Rabbit IgG and Alexa Fluor 594-labeled Goat Anti-Mouse IgG ZSGB-BIO Cat. No.: ZF-0516, ZF-0513) diluted in PBS were then incubated with the cells for 30–40 min at room temperature. DAPI staining was performed for 5 min, followed by mounting with an anti-fluorescence quenching mounting solution. Confocal microscopy was used to capture images of the cells using wavelengths of 405 nm, 561 nm, and 488 nm. The fluorescence intensity and expression of VP1, p-NF-κB p65, IL-1β and TNF-α were analyzed and quantified using the Image-Pro-Plus 6.0 image analysis system.

#### 2.6.4 Western blot detection of NF-κB and MAPK signaling pathway-related proteins

RD cells were treated with Quercetin and EV71 for 48 h, followed by harvesting and lysing in radioimmunoprecipitation assay (RIPA) lysis buffer (Beyotime Biotechnology, P0013C) containing a protease inhibitor cocktail. The lysates were centrifuged at 15,000 rpm for 15 min at 4°C. The protein concentration was determined using the bicinchoninic acid reagent (Beyotime Biotechnology Co., Ltd.), and the proteins were separated by sodium dodecyl sulfate-polyacrylamide gel electrophoresis. The electrophoresis products were then transferred to polyvinylidene fluoride membranes (Merck, Darmstadt, Germany). Subsequently, the membranes were incubated with primary antibodies in 5% BSA in TBST (TBS with 0.05% Tween-20) overnight at 4°C. Afterward, the membranes were washed three times with TBST for 10 min each and then incubated with secondary antibodies at 37°C for 1 h. After this, the membranes were washed three times with TBST for 10 min each time. Finally, chemiluminescent detection was performed using specific antibodies for the targeted proteins (ERK1/2, Santa Cruz Cat. No.: sc-514302; p-ERK, Santa Cruz Cat. No.: sc-7383; JNK, Santa Cruz Cat. No.: sc-7345; p-JNK, Santa Cruz Cat. No.: sc-6254; p38, Santa Cruz Cat. No.: sc-271120; p-p38, Santa Cruz Cat. No.: sc-7973; NF-κB p65, Santa Cruz Cat. No.: sc-515045, p-NF-κB p65, Santa Cruz Cat. No.: sc-135769). In the Western blotting analysis, it was observed that JNK exhibited strong signals at 54 kDa, with only a faint signal detected at 46 kDa. Similarly, ERK displayed strong signals at 42 kDa, with a weak signal at 44 kDa. This variance might be attributed to the antibodies’ specificity, prompting us to focus our analysis on the protein bands displaying strong signals.

### 2.7 Statistical analysis

The data analysis and visualization were performed using GraphPad Prism version 8.0 software (GraphPad Software, San Diego, CA, United States). The data are presented as mean ± SD. The data from *in vitro* experiments were analyzed using a one-way ANOVA analysis of variance, followed by the Tukey test for multiple comparison tests. A *p*-value of <0.05 was considered statistically significant.

## 3 Results

### 3.1 The active ingredients and target genes of ML

**FIGURE 1:**
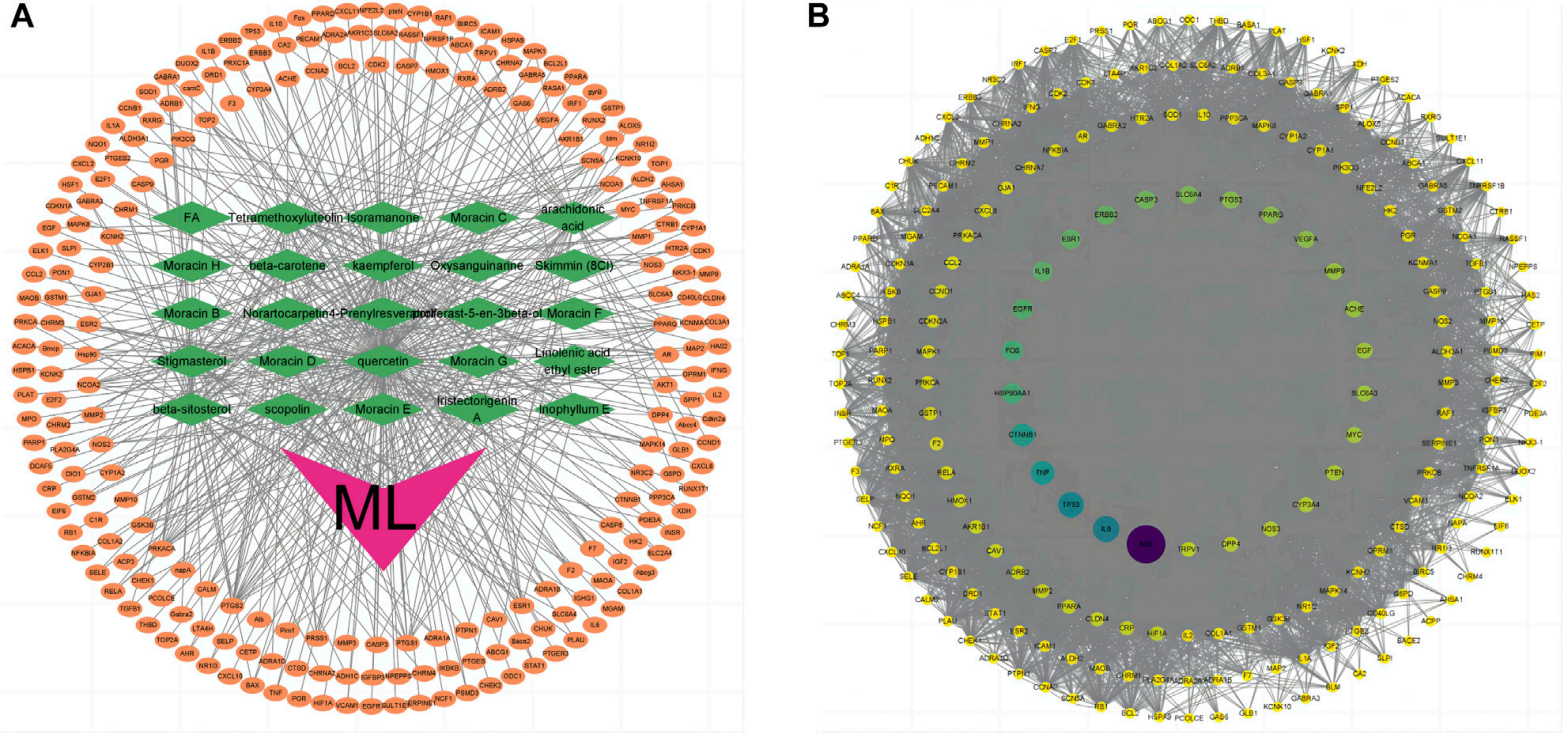
Target genes of ML. **(A)** “ML-active ingredient-target” network diagram. The orange oval in the outer circle of the figure is the target gene, the green diamond is the active ingredient of ML, and the purple “V” shape is the traditional Chinese medicine ML. **(B)** PPI network diagram of mulberry leaf-related genes. The larger the circle and the darker the color, the larger the node degree.

Utilizing the TCMSP database, we retrieved the active ingredients of ML, resulting in 269 unique compounds. Through a screening process, we identified 29 key active ingredients, and their respective target information was extracted from the database. The target information was then mapped to gene names using the UniProt database, resulting in 498 related target genes (Table 1). After removing duplicate entries, we obtained 231 potential target genes associated with ML. Using Cytoscape software, we constructed a network diagram to visualize the association between ML active ingredients and target genes (v3.9.1) (Figure 1A).

**TABLE 1:** ML’s active ingredients with anti-EV71 effects.

MOL ID	Compound name	OB	DL	Number of targets
MOL001771	poriferast-5-en-3beta-ol	36.91	0.75	2
MOL002218	scopolin	56.45	0.39	2
MOL002773	beta-carotene	37.18	0.58	22
MOL003842	Albanol	83.16	0.24	0
MOL003847	Inophyllum E	38.81	0.85	9
MOL003850	26-Hydroxy-dammara-20,24-dien-3-one	44.41	0.79	0
MOL003851	Isoramanone	39.97	0.51	3
MOL003856	Moracin B	55.85	0.23	7
MOL003857	Moracin C	82.13	0.29	6
MOL003858	Moracin D	60.93	0.38	14
MOL003859	Moracin E	56.08	0.38	11
MOL003860	Moracin F	53.81	0.23	2
MOL003861	Moracin G	75.78	0.42	4
MOL003862	Moracin H	74.35	0.51	4
MOL003879	4-Prenylresveratrol	40.54	0.21	17
MOL000433	FA	68.96	0.71	3
MOL000729	Oxysanguinarine	46.97	0.87	5
MOL000098	quercetin	46.43	0.28	154
MOL000358	beta-sitosterol	36.91	0.75	38
MOL000422	kaempferol	41.88	0.24	63
MOL000449	Stigmasterol	43.83	0.76	31
MOL001439	arachidonic acid	45.57	0.2	38
MOL001506	Supraene	33.55	0.42	0
MOL003759	Iristectorigenin A	63.36	0.34	22
MOL003975	icosa-11,14,17-trienoic acid methyl ester	44.81	0.23	0
MOL006630	Norartocarpetin	54.93	0.24	5
MOL007179	Linolenic acid ethyl ester	46.1	0.2	2
MOL007879	Tetramethoxyluteolin	43.68	0.37	32
MOL013083	Skimmin (8CI)	38.35	0.32	4

The presented table catalogs a series of active ingredients demonstrating anti-EV71 (Enterovirus 71) effects. Each row corresponds to a distinct compound. Compound name: This column contains the names of the active ingredients. OB (Oral Bioavailability): Oral bioavailability measures the extent to which an orally administered drug is absorbed into the systemic circulation. A higher numerical value indicates better absorption. DL (Drug-likeness): Drug-likeness shows a molecule’s potential to possess drug-like properties. A value closer to 1 suggests that the molecule has favorable drug-like attributes. The number of targets denotes how many target molecules each compound interacts with. In drug discovery, drugs often interact with multiple molecules to achieve their therapeutic effects.

### 3.2 The PPI network of mulberry leaf target genes was constructed

The relevant target genes associated with ML were obtained from the STRING protein database, specifically focusing on *Homo sapiens* species. The resulting protein-protein interaction (PPI) network diagram comprised 221 nodes and 4001 edges, representing mulberry leaf-related target genes. The average node degree in the network was 36.2. To visualize the network diagram, Cytoscape software (V3.9.1) was utilized, and the CytoNCA program package within the software was employed to arrange the graph based on node degree, determining the size and color of the nodes. The top 10 genes with the highest node degrees were *akt1, alb, il-6, tp53, tnf, ctnnb1, hsp90aa1, fos, egfr*, and *il-1b* (Figure 1B).

### 3.3 Potential target genes for anti-EV71 action of ML

**FIGURE 2:**
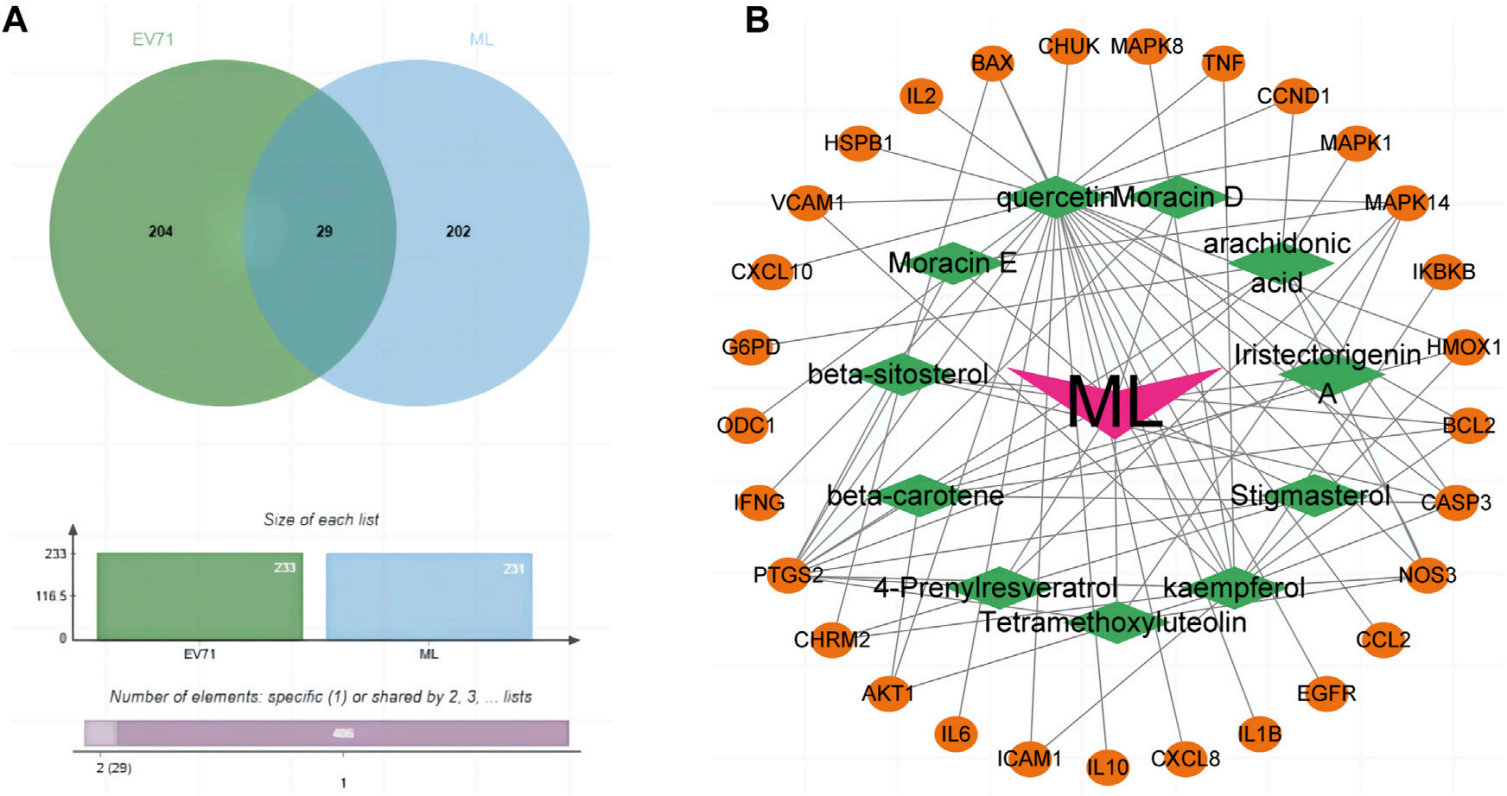
Prediction of potential anti-EV71 target genes in ML. **(A)** Venn diagram of disease drug targets. The green part is the target gene related to the EV71 virus, and the blue part is the target gene associated with ML. **(B)** Network diagram of common genes and active components of ML. The orange ellipse in the outer circle is the potential target gene of mulberry leaf resistance to EV71, the green diamond in the inner circle is the active ingredient of traditional Chinese medicine corresponding to these potential target genes, and the “V” shape in the middle is the traditional Chinese medicine mulberry leaf.

Through the GENECARDS and NCBI databases, a search with “EV71”as the query resulted in the obtaining of 233 EV71-related genes. These genes were then compared with the mulberry leaf-related target genes using the Jvenn online tool, resulting in 29 common genes (Figure 2A). We consider these 29 common genes as potential genes for ML in resisting the EV71 virus. The identified genes are: *hspb1, chrm2, odc1, akt1, il10, egfr, ikbkb, map, casp3, mapk1, chuk, vcam1, ifng, il6, cxcl8, ccl2, bcl2, ccnd1, bax, cxcl10, ptgs2, icam1, il1b, nos3, hmox1, mapk14, tnf, il2, and g6pd.*

### 3.4 Quercetin is a key component of ML in the fight against EV71

The prediction of potential target genes of ML against EV71 yielded 29 genes. Further screening was conducted to identify the active ingredients of ML associated with these target genes. Utilizing Cytoscape software (V3.9.1), a network diagram illustrating the interplay between the potential target genes and active ingredients of ML was generated (Figure 2B). Significantly, 25 out of the 29 common targets were found to be closely associated with Quercetin, highlighting its pivotal role as the critical component in ML for combating EV71.

### 3.5 Strong affinity between critical components of ML and EV71 target genes

**FIGURE 3:**
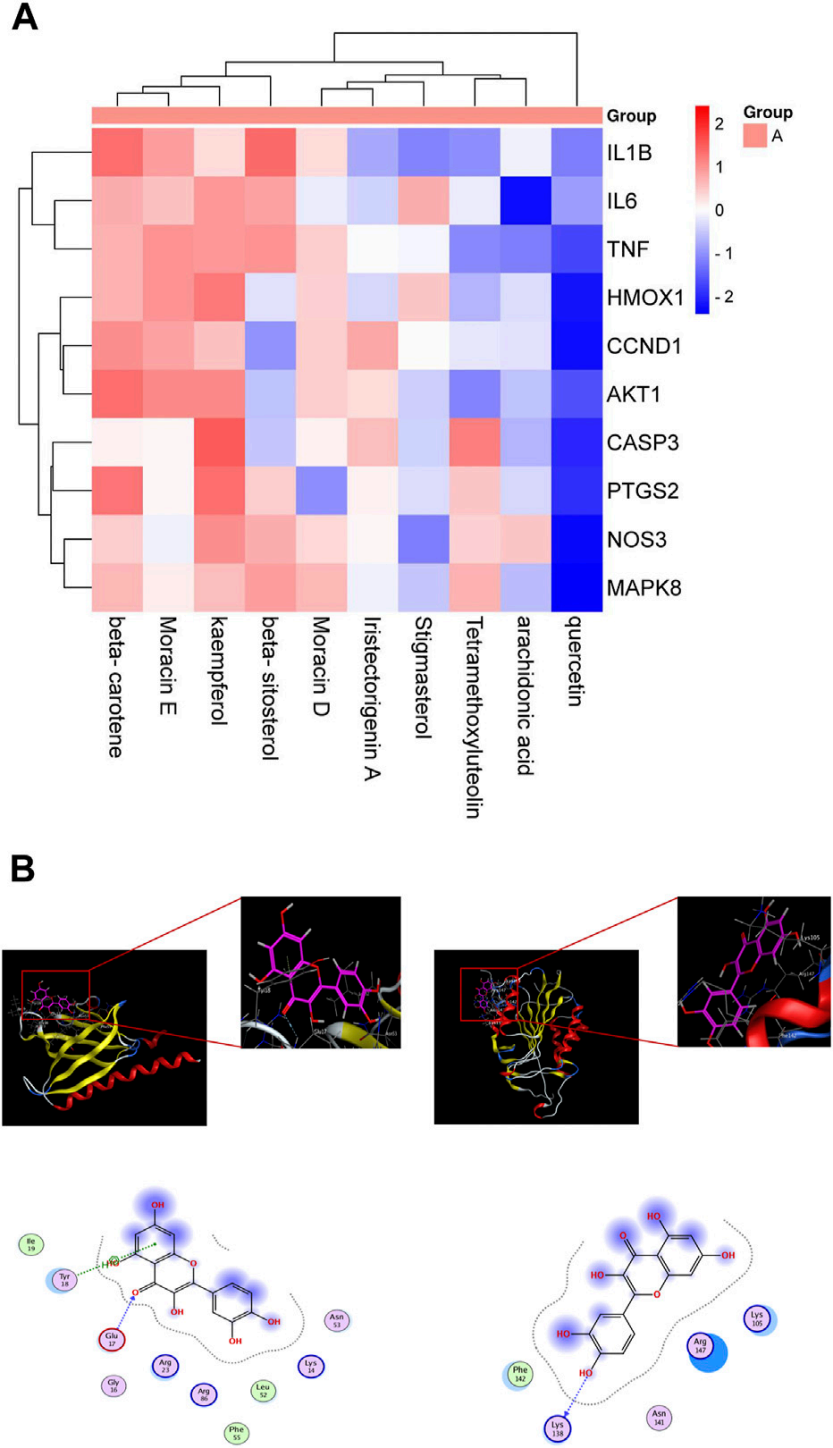
Molecular docking. **(A)** Docking and binding ability of target gene and small molecule library molecule. **(B)** Molecular docking model. Red is alkyl conjugation, and green is van der Waals interaction.

We employed the String database to import twenty-five common targets of Quercetin and EV71. Meticulous analysis successfully identified the top ten interacting genes: *akt1, ccnd1, ptgs2, tnf, casp3, hmox1, mapk8, il1b, il6,* and *nos3*. Our objective was to pinpoint key components in ML that hold potential therapeutic efficacy against EV71. Ten key ingredients were selected from the potential components of ML against EV71: beta-sitosterol, arachidonic acid, tetramethoxyluteolin, Moracin D, Quercetin, beta-carotene, stigmasterol, kaempferol, Moracin E, and iristectorigenin A. These active ingredients were organized into a small molecule library, and molecular docking was performed with the top ten target genes to obtain binding energy values. A heatmap was generated to display the binding energy results (Figure 3A). The results demonstrated that the binding energies between the target genes and the active components in the small molecule library were all ≤ −4.25 kcal mol^−1^, indicating a strong interaction between the main active ingredients of ML and the macromolecular protein against EV71, specifically involving key residues. Quercetin exhibits a remarkable affinity towards the target mentioned above genes, thus enabling us to conduct a preliminary assessment of its efficacy in combating EV71. The results with strong binding ability were visualized using MOE 2019.0102 software (Figure 3B).

### 3.6 NF-κB signaling pathway is the primary mechanism of Quercetin anti-EV71

**FIGURE 4:**
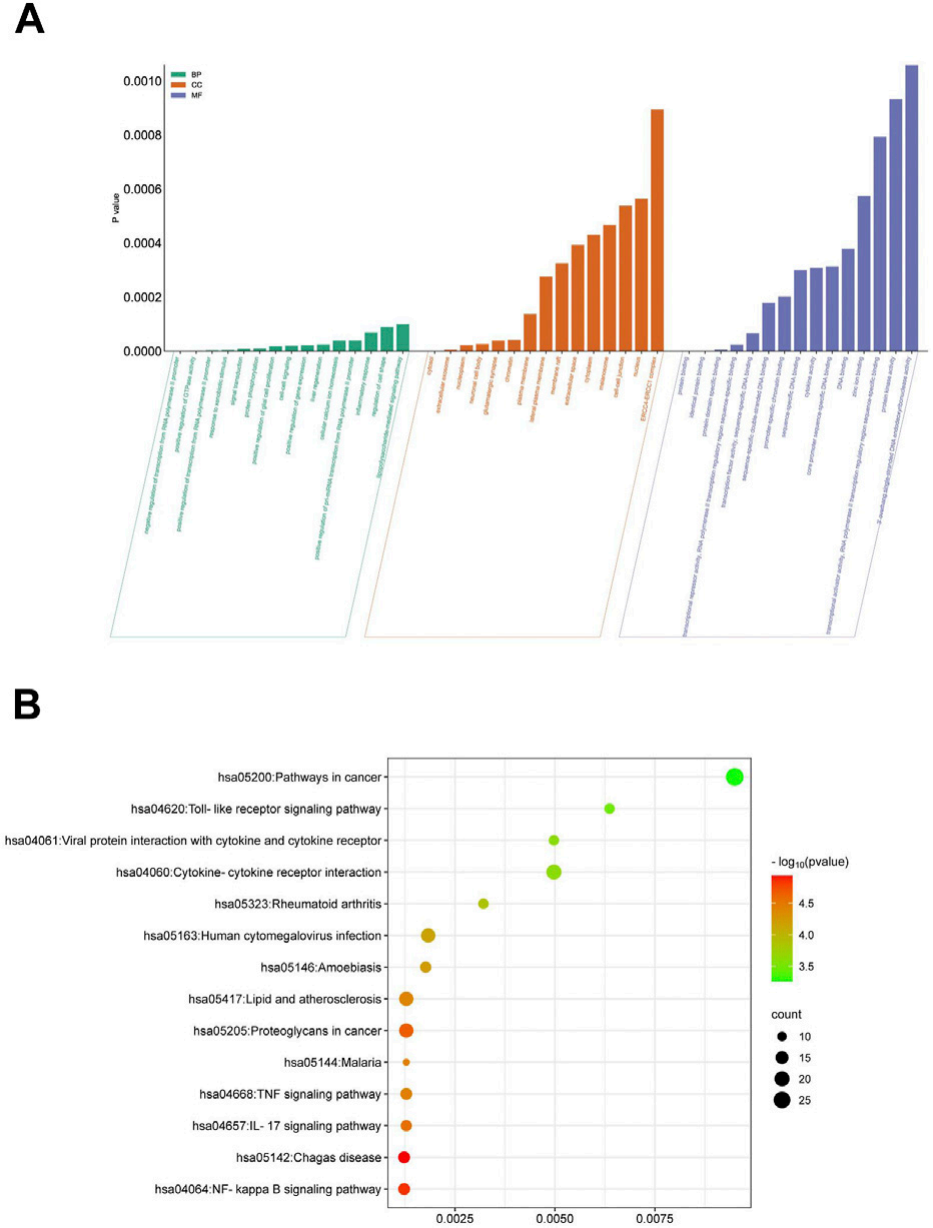
GO and KEGG enrichment analysis. **(A)** Functional enrichment of GO molecules. **(B)** KEGG metabolic pathway enrichment analysis.

Quercetin assumes a pivotal role in the efficacy of ML against EV71. We conducted an in-depth analysis to identify 25 target genes associated with Quercetin’s action against EV71, followed by performing an enrichment analysis utilizing the DAVID database. This analysis yielded 402 enriched Gene Ontology (GO) items, encompassing 268 biological processes (BP), 75 cellular components (CC), and 59 molecular functions (MF) (Figure 4A). Additionally, KEGG pathway analysis identified 66 enriched signaling pathways (Figure 4B). We selected the top 15 items based on their *p* values to investigate these findings further and visualized them using R 4.2.1 software. The enriched terms encompass diverse functions, including negative regulation of transcription from RNA polymerase II promoter, positive regulation of GTPase activity, cytosol, extracellular exosome, protein binding, and identical protein binding. In parallel, KEGG analysis revealed relevant pathways such as the NF-κB signaling pathway, IL-17 signaling pathway, and TNF signaling pathway. These comprehensive findings strongly suggest that Quercetin, the primary active ingredient in ML, may exert its antiviral effect against EV71 through involvement in these signaling pathways, mainly via the NF-κB signaling pathway.

### 3.7 Quercetin improves survival of EV71-infected RD cells by inhibiting the NF-κB signaling pathway

**FIGURE 5:**
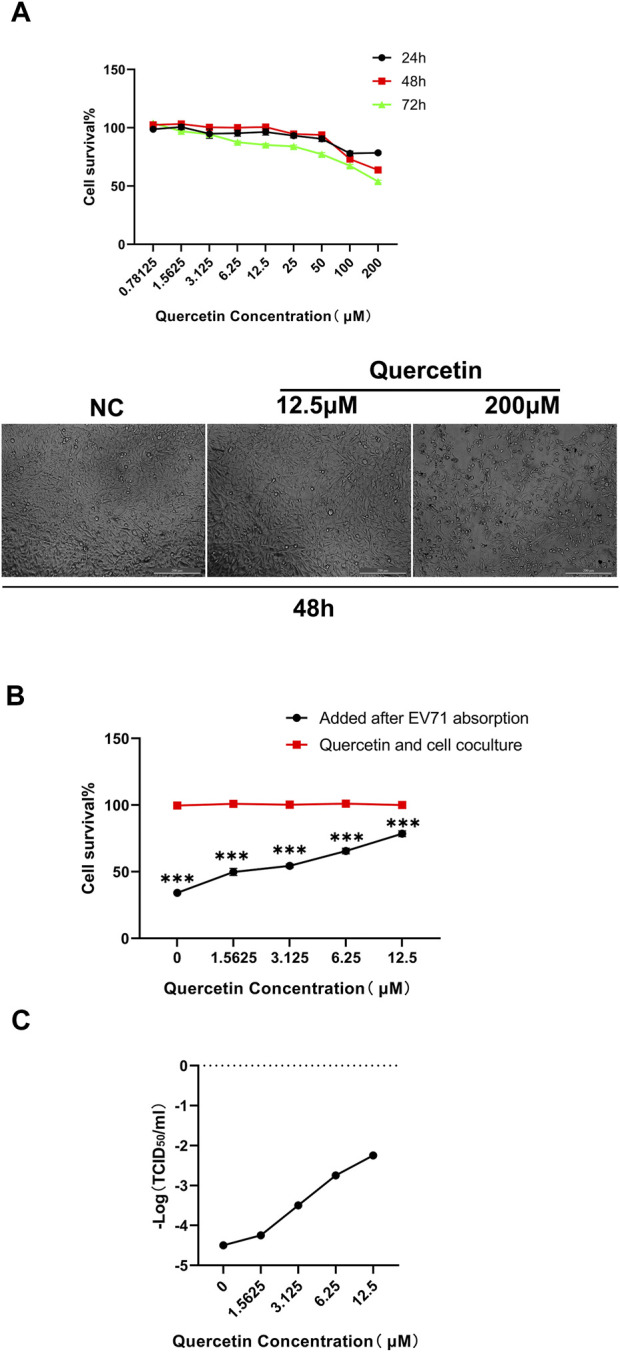
Quercetin improves survival of EV71-infected RD cells. **(A)** Detection of Quercetin on RD cytotoxicity. Quercetin was serially diluted in media containing 2% FBS at concentrations of 0, 0.78125, 1.5625, 3.125, 6.25, 12.5, 25, 50, 100, and 200 μM, and no quercetin was considered as a normal RD cell control. Subsequently, the cytotoxicity of Quercetin on RD cells was determined by the CCK-8 assay. Measure the absorbance at 450 nm using a microplate reader. The TC_0_ value was calculated as the maximum non-toxic concentration of the drug. Data are presented as mean values from three independent experiments. **(B)** Inhibitory effect of Quercetin on EV71 replication. Quercetin was diluted in DMEM with 2% FBS to 0, 1.5625, 3.125, 6.25 and 12.5 μM. No quercetin was considered in the EV71-infected control group, and the antiviral effect of Quercetin was tested. Data are represented as means from three independent experiments, SD, and analyzed by one-way ANOVA, compared with the NC group (***p < 0.001). **(C)**Inhibitory effect of quercetin on EV71 virus TCID50. RD cells were infected with EV71 and treated with Quercetin. The replication ability of EV71 was significantly weakened upon quercetin treatment. These findings highlight the potent inhibitory effect of Quercetin on EV71 virus replication.

The CCK-8 assay was conducted to assess the cytotoxicity of different concentrations of Quercetin on RD cells at 24 h, 48 h, and 72 h. It was determined that the maximum non-toxic concentration (TC_0_) of Quercetin on RD cells was 12.5 μM (Figure 5A). Additionally, we employed GraphPad Prism version 8.0 software to compute the drug’s EC50 (78.69 μM) and IC50 (291.2 μM) at the 48-h mark. Subsequently, we determined the selection index (SI) to be 3.7. The cytopathic effects of the virus on RD cells were observed, and the TCID_50_ of the EV71 virus was calculated using the Reed-Muench method as 10^−4.5^/mL. For subsequent experiments, a concentration of Quercetin below TC_0_ (12.5 μM) and 100 TCID_50_ (10^−2.5^/mL) of EV71 virus were selected for a 48-h assay.

The effectiveness of different concentrations of Quercetin against the EV71 virus was assessed using the CCK-8 method. The results revealed that 12.5 M Quercetin exhibited the most significant anti-EV71 virus effect. Compared to the cell control group (100% survival rate), the survival rates were 53.86% at 25 μM, 76.11% at 12.5 μM, 63.20% at 6.25 μM, 52.82% at 3.125 μM, 47.02% at 1.5625 μM, and 35.51% in the virus group (Figure 5B). The TCID50 detection results demonstrated a notable decrease in the virus replication capability of EV71 treated with Quercetin compared to RD cells infected with regular EV71. This observation exhibited a correlation with the varying concentrations of Quercetin utilized (Figure 5C).

**FIGURE 6:**
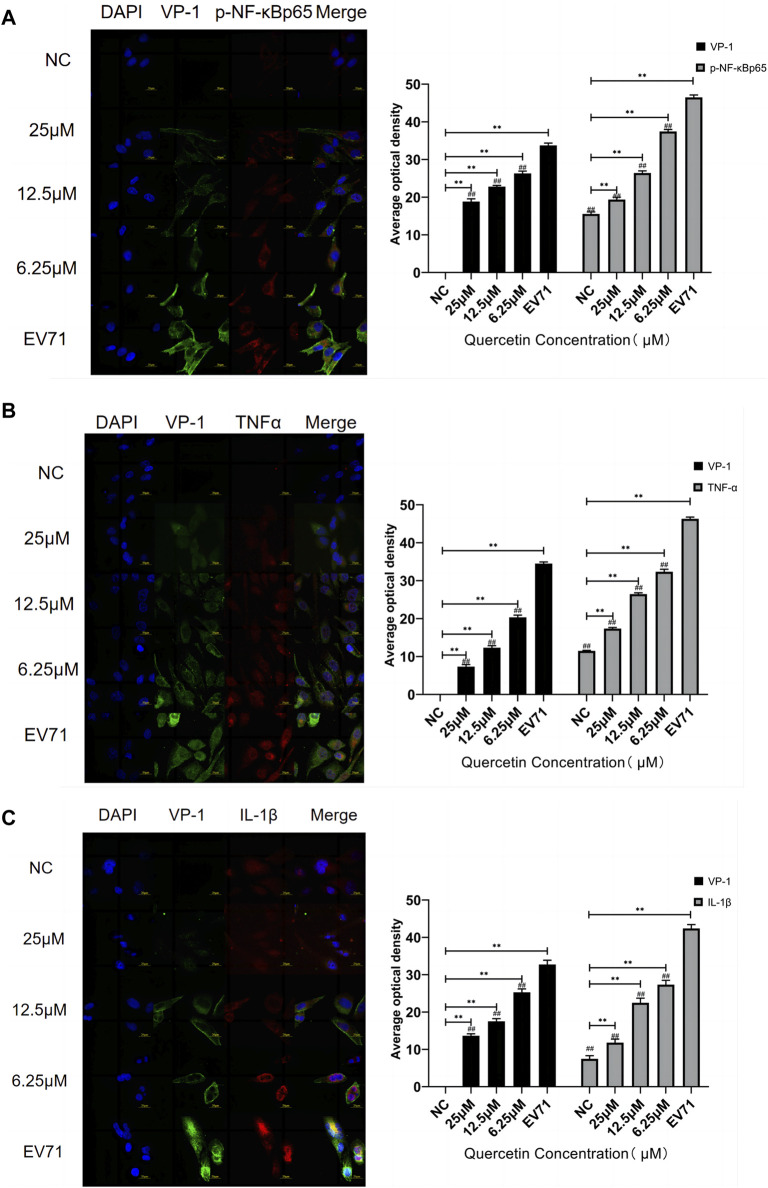
Quercetin inhibited the co-localization staining of NF-κB signaling pathway-related proteins and VP-1 in EV71-infected RD cells. **(A)** VP-1 and p-NF-κB p65 protein levels in RD cells. **(B)** VP-1 and TNFα protein levels in RD cells. **(C)** VP-1 and IL-1β protein levels in RD cells. (immunofluorescence, 600×, scale bar: 20 μm). Comparison with normal control group, **p < 0.01. Comparison with EV71-infected group ##*p* < 0.01.

**FIGURE 7:**
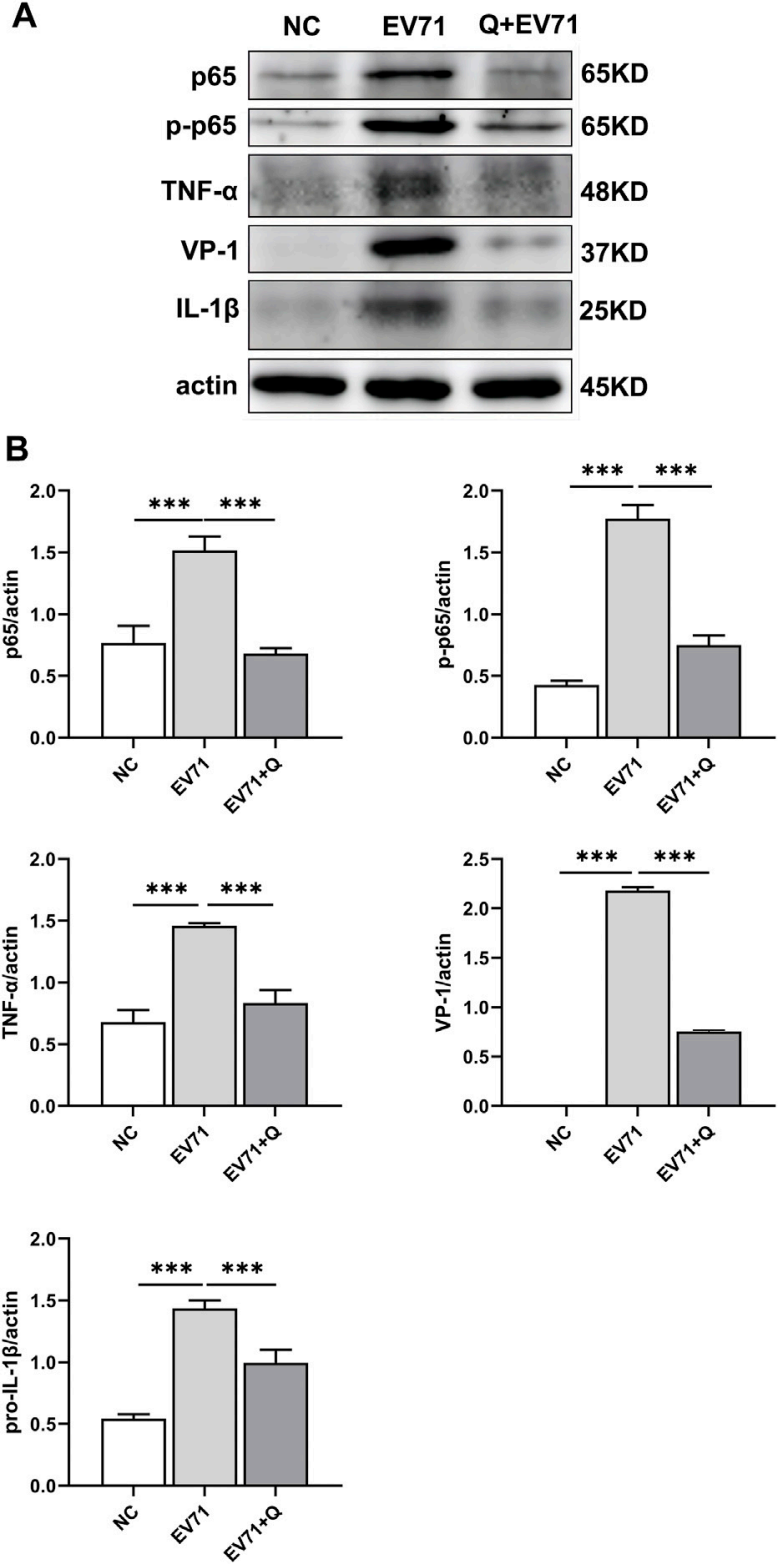
Quercetin inhibited activation of the NF-κB signaling pathway in EV71-infected RD cells. **(A)** Western blot analysis was performed to evaluate the effect of Quercetin on the expression of proteins related to the NF-κB signaling pathway in EV71-infected RD cells. It was observed that EV71 infection triggered activation of the NF-κB signaling pathway. However, subsequent treatment with Quercetin resulted in attenuated activation of NF-κB. **(B)** After actin normalization, we assessed the ratio of related proteins within the NF-κB signaling pathway. Our statistical analysis showed that compared with the EV71 group, the difference was statistically significant (*p < 0.05, **p < 0.01, ***p < 0.001).

VP1 of the EV71 virus was co-stained with p-NF-κBp65, TNF, and IL-1β. Co-localization of VP1 with p-NF-κBp65, TNF, and IL-1β proteins was observed. The levels of p-NF-κBp65, TNF, and IL-1β were significantly higher compared to the control group. In the quercetin treatment group, p-NF-κBp65, TNF, and IL-1β levels showed a dose-dependent reduction (Figure 6). These findings suggest that the anti-EV71 virus mechanism of Quercetin may involve the inhibition of the NF-κB signaling pathway. The Western blot results corroborated the findings observed in the Immunofluorescence analysis (Figure 7).

### 3.8 Quercetin inhibits the MAPK signaling pathway

**FIGURE 8:**
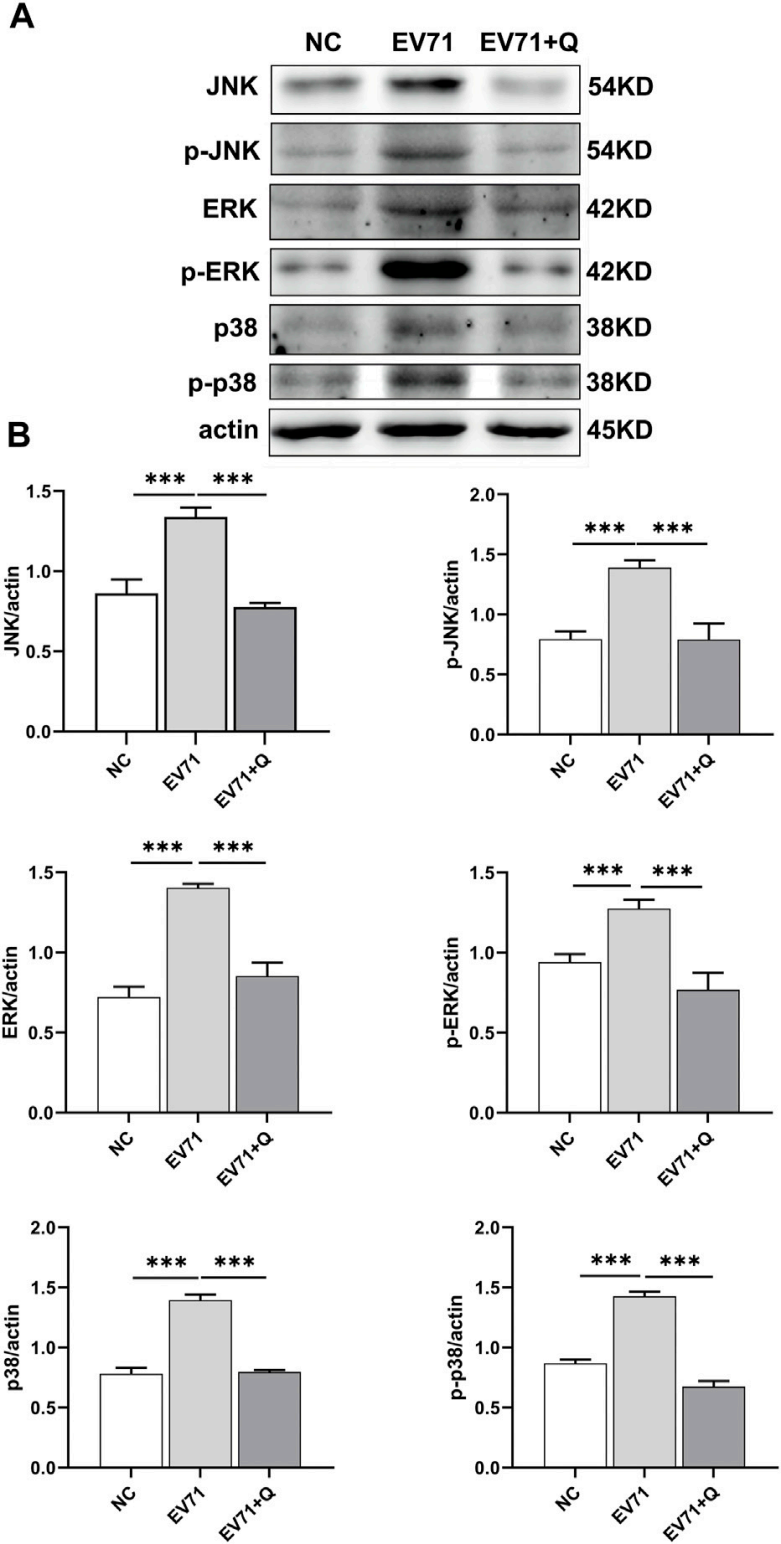
Quercetin inhibits the activation of the MAPK signaling pathway in EV71-infected RD cells. **(A)** Using Western blot analysis, we assessed the effect of Quercetin on the expression of proteins within the MAPK signaling pathway in EV71-infected RD cells. Our findings suggest that EV71 infection triggers the activation of the MAPK signaling pathway. Subsequent quercetin treatment produced results consistent with the inhibitory effects observed in the NF-κB signaling pathway. **(B)** After actin normalization, we assessed the proportion of associated proteins within the MAPK signaling pathway. Our statistical analysis showed that compared with the EV71 group, the difference was statistically significant (*p < 0.05, **p < 0.01, ***p < 0.001).

JNK, ERK, and p38 are proteins related to the MAPK signaling pathway. The Western blotting results showed that the EV71 virus could cause overactivation of the MAPK pathway in RD cells, with increased expression and phosphorylation levels of JNK, ERK, and p38. Quercetin demonstrated a downregulation in the expression and inhibition of JNK, ERK, and p38 phosphorylation caused by EV71. Quercetin inhibited the MAPK signaling pathway activated by EV71 (Figure 8).

## 4 Discussion

HFMD is a highly contagious viral illness primarily caused by enteroviruses, notably coxsackievirus A16 (CV-A16) and enterovirus 71 (Aswathyraj et al., 2016; Xing et al., 2014). Among them, EV71 is associated with the most severe symptoms and highest mortality rate (Yang et al., 2009; Zhang et al., 2010; Li et al., 2017). Unfortunately, no specific drugs are currently available for the treatment of these viruses (Ng et al., 2015). Extensive research efforts have been dedicated to developing anti-EV71 drugs over the past 50 years (Wang et al., 2023). Traditional Chinese medicine has revealed promising monomers, such as glycyrrhizic acid, baicalin, indigo root, Quercetin, kaempferol, and apigenin, which possess anti-EV71 effects (Tsai et al., 2011; Yang et al., 2012; Wang et al., 2013; Zhang et al., 2014; Li et al., 2015; Wang et al., 2016; Yao et al., 2018; Dai et al., 2019). Some of these components are already utilized in clinical practice. While Western medicines exhibit satisfactory inhibitory effects on EV71 replication, their mechanisms often target a single aspect and carry a risk of carcinogenesis (Friedman et al., 2009; Brambilla et al., 2010). In contrast, traditional Chinese medicine embraces a holistic approach, exerting antiviral effects through overall balance. Chinese medicine is distinguished by its multifaceted components and targets, affordability, low toxicity, and minimal cancer risk (Csikós et al., 2021; Zhu et al., 2022). Its antiviral properties are achieved through antioxidant, immune regulatory effects (Colunga Biancatelli et al., 2020), inhibition of viral-induced inflammatory responses (Lim et al., 2018; Saeedi-Boroujeni and Mahmoudian-Sani, 2021), and suppression of virus replication by inhibiting oxidative stress (Chen et al., 2022).

ML, a traditional Chinese medicine, has significant medicinal value and has shown efficacy in treating metabolic disorders such as diabetes, dyslipidemia, obesity, atherosclerosis, and hypertension (Zhang et al., 2022; Cheng et al., 2022). Furthermore, ML has been identified as a potential treatment option for viral diseases (Pronin et al., 2021). Our research was primarily dedicated to unraveling the antiviral attributes of mulberry leaves (ML), identifying multiple active constituents with potent antiviral effects. An extensive body of research has effectively showcased the antiviral prowess of compounds such as Quercetin, kaempferol, and beta-carotene (Sheehan et al., 2012; Ding et al., 2018; Shen et al., 2021; Chen et al., 2022; Shokry et al., 2023). Furthermore, stigmasterol has demonstrated its multifaceted potential, encompassing antioxidant, antiviral, antifungal, antibacterial, and anticancer properties, achieved through immune regulation and anti-inflammatory mechanisms (Petrera et al., 2014). Iristectorigenin A possesses antioxidant and anti-inflammatory effects (Al-Qudah et al., 2015; Lim et al., 2017). Moracin D and Moracin E exhibit antioxidant effects and hold substantial medicinal value in antiviral and anticancer research (Yoon et al., 2021; Mohan Kumar et al., 2022). These active constituents of ML exert antiviral effects through diverse mechanisms, highlighting the potential of ML in countering the EV71 virus. Furthermore, the collective antiviral impacts of these active constituents might demonstrate synergistic properties.

By examining 29 common targets shared by ML and EV71, we predicted the potential function of these genes in ML anti-E71 virus through network pharmacology studies. A number of these genes are involved in oxidative stress, inflammation, vascular permeability, and immune function. For example, *AKT1* activation promotes cell proliferation and suppresses cell apoptosis, making it a significant participant in EV71’s immune-inflammation mechanism (Shi et al., 2013). *IL-6, IL-1B,* and *TNF-α* exhibit immunomodulatory and pro-inflammatory effects (Luo et al., 2019; Yi et al., 2020). *MAPK1* is crucial in the inflammatory response (Xu et al., 2023). Inhibiting *CASP3* activity reduces EV71 virus protein expression and replication and can trigger pyroptosis as an alternative to apoptosis, thereby hindering EV71 infection (Song et al., 2018). *HMOX1* contributes to the host’s resistance to the virus through the oxidative stress defense system (Zhang et al., 2022). In summary, these target genes of ML employ diverse mechanisms to inhibit EV71 virus infection and exert their influence at various stages of disease development.

To elucidate the mechanism of ML against the EV71 virus, we conducted GO enrichment analysis on the key targets of Quercetin, the primary anti-EV71 component of ML, and performed KEGG enrichment analysis to predict associated signaling pathways responsible for the antiviral effect. The KEGG database encompasses diverse biological domains, furnishing extensive data and insights spanning genomics, proteomics, metabolomics, and more. This encompassing repository facilitates the identification of promising targets associated with drugs and ailments, along with pertinent signaling pathways and biological functions (Lu, et al., 2020). Rigorous procedures have been implemented to ascertain the dependability of our KEGG enrichment analysis outcomes. In this process, data sources are carefully reviewed, statistical significance is evaluated, cross-validation is carried out thoroughly, and the biological relevance of the data is carefully interpreted. The findings suggest a potential relationship between Quercetin and the NF-κB signaling pathway. This pathway, comprising canonical and non-canonical pathways, plays a vital role in various biological processes, including the regulation of B and T-cell immunity (Lu et al., 2021). Many viruses activate or evade antiviral immune responses through this pathway (Struzik and Szulc-Dąbrowska, 2019; Khatiwada et al., 2017). EV71 triggers NLRP3 inflammasome activation via the NF-κB pathway, and inhibiting this pathway aids the host’s defense against EV71 infection in the central nervous system (Gong et al., 2022). Severe EV71 infection is associated with significantly elevated TNF levels (Duan et al., 2014; Sun et al., 2018). The TNF-α-mediated NF-κB pathway is essential for inflammatory responses, and the 2C protein of EV71 promotes NF-κB activation via TNF-α. The activation of NF-κB can be triggered by the TNF-α-related factor 2, the MEK kinase 1, the IKKα, or the IKKβ (Zheng et al., 2011).

To validate the antiviral effect and mechanism of ML, we conducted *in vitro* experiments to verify the antiviral activity of Quercetin, the primary active compound derived from ML. Immunofluorescence analysis was performed to assess the expression of essential proteins in the NF-κB signaling pathway. The results demonstrated that Quercetin exhibited significant inhibition against the EV71 virus. Pretreatment of the virus with Quercetin weakened its toxicity and directly killed it, leading to its antiviral effect in cell experiments. Immunofluorescence analysis revealed reduced protein levels of p-NF-κB p65, TNF-α, and IL-1β in the quercetin-treated group compared to the EV71 group. The NF-κB signaling pathway is crucial in the inflammatory response (Oeckinghaus et al., 2011). TNF-α activates the non-canonical NF-κB pathway, leading to an inflammatory response (Yu et al., 2020). Downstream kinase IKKα is activated by TNF-α, promoting NF-κB phosphorylation (Sun, 2011). IL-1β induces NF-κB inhibitor phosphorylation, translating NF-κB to the nucleus and transcription of cytokine and chemokine genes (Cheng et al., 2019). The mammalian NF-κB family comprises five distinct proteins: p50, p52, p65 (also recognized as RelA), RelB, and c-Rel. These NF-κB family constituents engage in diverse combinations of homo- and heterodimeric associations with each other, culminating in the formation of biologically active protein complexes. Among these, the p65-p50 heterodimer is the prevailing form within cellular contexts (Chen, et al., 1998). Notably, the p65 protein garners substantial attention among the five NF-κB family members due to its extensive scrutiny. This heightened focus is partly attributable to its role as an activating component within the p65-p50 heterodimeric complex (Lecoq, et al., 2017). Upon infection with a pathogen, the activation of the predominant p65-p50 heterodimer of NF-κB occurs, leading to the translocation of p65 and p50 into the nucleus (Medina et al., 2002). Additionally, the phosphorylation of IκBα, mediated by IκB induced by TNF-α, results in its ubiquitination. This process ultimately leads to the nuclear translocation of NF-κB and the regulation of target gene transcription (Papa et al., 2009). The nuclear translocation of NF-κB p65 and NF-κB p50 has been observed in the mouse liver following infection with the Dengue virus (DENV) (Sreekanth et al., 2020). This study investigated p65 and phosphor-p65 in the cytoplasm and nucleus of whole-cell lysates. Separate studies on these fractions would provide more insight into Quercetin’s role in regulating nuclear translocation of p65. The inhibitory effect of Quercetin on the NF-κB signaling pathway and its ability to decrease the synthesis of pro-inflammatory cytokines, specifically TNF-α and IL-1β, as observed in the study conducted by (Bezzi et al., 2001), aligns with the findings of the current investigation, suggesting that this mechanism may contribute significantly to Quercetin’s antiviral activity against EV71.

Moreover, our investigation into the conduction of the MAPK signaling pathway unveiled that Quercetin’s impact on this pathway parallels that of the NF-κB signaling pathway. Notably, the inhibitory effect of Quercetin on both pathways follows a dose-dependent pattern. The MAPK signaling pathway employs at least three activation routes to transmit extracellular signals to the nucleus. These include the classical MAPK pathway, MAPK/ERK, the JNK/MAPK signaling pathway, and the p38/MAPK signaling pathway. The MAPK/ERK signaling pathway is activated by signals from cell surface receptors like receptor tyrosine kinases (RTKs) or G protein-coupled receptors (GPCRs) (Delire and Stärkel, 2015). The process of ERK activation entails phosphorylation by activated RAF, subsequently activating MEK, resulting in the direct phosphorylation of ERK (Liu et al., 2018). It has been documented that garlic extract inhibits reticuloendotheliosis virus (REV) replication by suppressing ERK expression (Wang et al., 2017). DNA viruses, including Herpes Simplex Virus 1 (HSV-1), exploit the MAPK/ERK pathway for intercellular dissemination (DuShane and Maginnis, 2019; Watanabe et al., 2021). JNK, also recognized as stress-activated protein kinase (SAPK), represents a subfamily within the canonical MAPK signal transduction cascade (Zeke, et al., 2016; Pua, et al., 2022). JNK proteins promptly respond to various cellular stimuli, including inflammatory cytokines, growth factors, ultraviolet radiation, bacterial and viral infections, heat shock, and osmotic and genotoxic stress (Kusumaningrum, et al., 2018). The promotion of Duck Plague Virus (DPV) proliferation has been documented through the inhibition of the immune interferon (IFN) signaling pathway and inflammatory pathways by JNK, as reported by Wu, et al. (2022). Furthermore, the activation of JNK plays a crucial role in Varicella-Zoster Virus (VZV) protein expression and replication, as highlighted by Kurapati et al. (2017). p38 mitogen-activated protein kinases form a class of evolutionarily conserved serine/threonine kinases. They function as intermediaries, connecting extracellular signals to intracellular processes that regulate a multitude of cellular functions (Falcicchia, et al., 2020; Romero-Becerra, et al., 2020). Various extracellular stimuli can phosphorylate p38 through the classical MAPK kinase (MAP3K)–MAP kinase kinase (MKK) pathway. Phosphorylated p38, in turn, activates an array of transcription factors, protein kinases, cytoplasmic and nuclear proteins, and substrates downstream of this phosphorylation encompass the regulation of inflammatory responses, cell differentiation, apoptosis, and more (Yao, et al., 2020; García-Hernández, et al., 2021; O'Neil, et al., 2018). Indeed, recent studies have demonstrated that infection with severe acute respiratory syndrome coronavirus 2 (SARS-CoV-2) can induce the activation of p38/MAPK, resulting in an upregulation of proinflammatory cytokines and an enhanced replication of the virus (Bouhaddou et al., 2020). It has been observed that inhibiting the p38/MAPK signaling pathway can effectively mitigate the Influenza virus (IV) replication and the excessive production of proinflammatory mediators (Yang et al., 2022). Additionally, the infection of Newcastle Disease Virus (NDV) has been shown to induce the activation of p38/MAPK/Mnk1 signaling, facilitating the efficient synthesis of viral proteins (Zhan et al., 2020). Previous research has indicated that inhibiting the MAPK signaling pathway has significant implications in the context of viral infections (Sun et al., 2023). The infection caused by EV71 is closely linked to the signaling pathways of JNK and p38 MAPK, which in turn activate the MAPK pathway, increasing virus production and releasing proinflammatory cytokines (Peng et al., 2014). EV71 triggers the activation of the ERK MAPK pathway through the induction of c-Src-mediated epidermal growth factor receptor (EGFR) activation (Wong et al., 2005; Tung et al., 2011). Inflammation occurs as a result of the activation of the MAPK pathway downstream of the NF-κB signaling pathway (Roth Flach et al., 2015; Ramalingam et al., 2020). In contrast, an overabundance of phosphorylation in downstream proteins of MAPK has the potential to induce the release and nuclear translocation of NF-κB, thereby intensifying the inflammatory response (Schulze-Osthoff et al., 1997; Papa et al., 2009; Sreekanth et al., 2020; Liu et al., 2022). Our research findings underscore that Quercetin orchestrates the modulation of the inflammatory response through these three MAPK pathways. This influence encompasses the phosphorylation of p65 by inhibiting the MAPK signaling pathway. Quercetin further curtails the inflammatory response by inhibiting the NF-κB signaling pathway, thus presenting a multifaceted mechanism for mitigating inflammation.

## 5 Conclusion

In conclusion, this study employed network pharmacology and *in vitro* experiments to investigate the mechanism of action of ML against EV71 virus infection. The findings reveal that ML exerts a substantial pharmacological effect against EV71 virus infection through a multi-component, multi-target, and multi-pathway approach. Quercetin, as the primary active component of ML, plays a pivotal role in inhibiting EV71 virus infection by targeting the NF-κB signaling pathway. These results provide valuable insights into the therapeutic mechanism of ML in the treatment of EV71 virus infection.

## Data Availability

The original contributions presented in the study are included in the article. Further inquiries can be directed to the corresponding authors.
