# Corrigendum: Network pharmacology-based exploration identified the antiviral efficacy of Quercetin isolated from mulberry leaves against enterovirus 71 via the NF-κB signaling pathway

**DOI:** 10.3389/fphar.2024.1421483

**Published:** 2024-07-08

**Authors:** Tianrun Liu, Yingyu Li, Lumeng Wang, Xiaomeng Zhang, Yuxuan Zhang, Xuejie Gai, Li Chen, Lei Liu, Limin Yang, Baixin Wang

**Affiliations:** ^1^ School of Medicine, Jiamusi University, Jiamusi, China; ^2^ The Affiliated First Hospital, Jiamusi University, Jiamusi, China; ^3^ School of Medicine, Dalian University, Dalian, China

**Keywords:** mulberry leaves, Quercetin, network pharmacology, enterovirus type 71, NF-kB

In the published article, there was an error in Figure 6 as published. The images of Figure 6A, C were duplicated. The corrected Figure 6 and its caption appear below.

**FIGURE 6:**
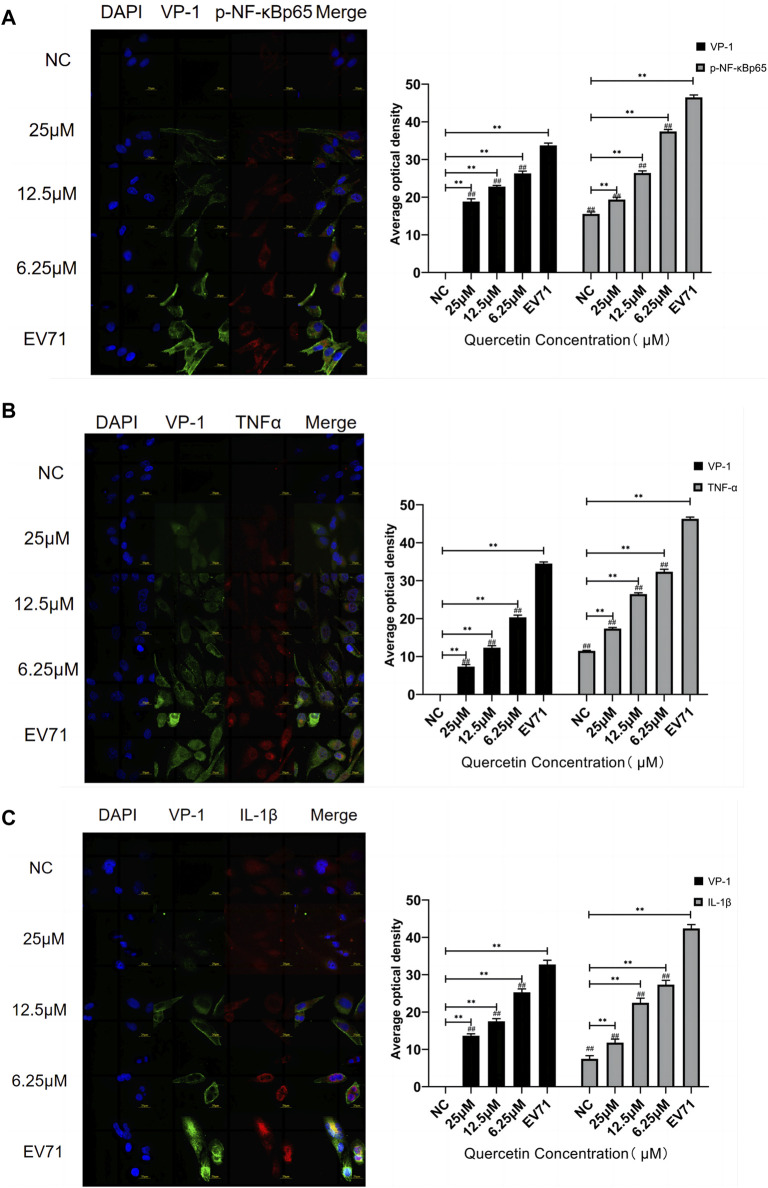
Quercetin inhibited the co-localization staining of NF-κB signaling pathway-related proteins and VP-1 in EV71-infected RD cells. **(A)** VP-1 and p-NF-κB p65 protein levels in RD cells. **(B)** VP-1 and TNF-α protein levels in RD cells. **(C)** VP-1 and IL-1β protein levels in RD cells. (Immunofluorescence, 600×, scale bar: 20 μm). Comparison with normal control group, **p < 0.01. Comparison with EV71-infected group ##*p* < 0.01.

The authors apologize for this error and state that this does not change the scientific conclusions of the article in any way. The original article has been updated.

